# Liver transcriptome response to hyperthermic stress in three distinct chicken lines

**DOI:** 10.1186/s12864-016-3291-0

**Published:** 2016-11-22

**Authors:** Xi Lan, John C. F. Hsieh, Carl J. Schmidt, Qing Zhu, Susan J. Lamont

**Affiliations:** 1 Farm Animal Genetic Resources Exploration and Innovation Key Laboratory of Sichuan Province, Sichuan Agricultural University, Chengdu Campus, Sichuan Province, China; 2 Department of Animal Science, Iowa State University, Ames, IA USA; 3 Department of Animal and Food Sciences, University of Delaware, Newark, DE USA

**Keywords:** Heat stress, Chicken, Liver, Transcriptome

## Abstract

**Background:**

High ambient temperatures cause stress in poultry, especially for broiler lines, which are genetically selected for rapid muscle growth. RNA-seq technology provides powerful insights into environmental response from a highly metabolic tissue, the liver. We investigated the effects of acute (3 h, 35 °C) and chronic (7d of 35 °C for 7 h/d) heat stress on the liver transcriptome of 3-week-old chicks of a heat-susceptible broiler line, a heat-resistant Fayoumi line, and their advanced intercross line (AIL).

**Results:**

Transcriptome sequencing of 48 male chickens using Illumina HiSeq 2500 technology yielded an average of 33.9 million, 100 base-pair, single-end reads per sample. There were 8 times more differentially expressed genes (DEGs) (FDR < 0.05) in broilers (*n* = 627) than Fayoumis (*n* = 78) when comparing the acute-heat samples to the control (25 °C) samples. Contrasting genetic lines under similar heat treatments, the highest number of DEGs appeared between Fayoumi and broiler lines. Principal component analysis of gene expression and analysis of the number of DEGs suggested that the AIL had a transcriptomic response more similar to the Fayoumi than the broiler line during acute heat stress. The number of DEGs also suggested that acute heat stress had greater impact on the broiler liver transcriptome than chronic heat stress. The angiopoietin-like 4 (ANGPTL4) gene was identified as differentially expressed among all 6 contrasts. Ingenuity Pathway Analysis (IPA) created a novel network that combines the heat shock protein family with immune response genes.

**Conclusions:**

This study extends our understanding of the liver transcriptome response to different heat exposure treatments in distinct genetic chicken lines and provides information necessary for breeding birds to be more resilient to the negative impacts of heat. The data strongly suggest ANGPTL4 as a candidate gene for improvement of heat tolerance in chickens.

**Electronic supplementary material:**

The online version of this article (doi:10.1186/s12864-016-3291-0) contains supplementary material, which is available to authorized users.

## Background

Climate change negatively impacts animals, resulting in significant welfare concerns and economic losses in livestock industries [1, 2]. Chicken is the second most popular food animal globally, producing high-quality protein with a low proportion of fat and playing a vital role in sustaining the world’s food production. Heat stress causes an estimated economic loss of more than one-hundred million dollars annually for the US poultry industry. Heat stress is especially taxing for rapidly growing meat-type chickens (broilers), causing detrimental impacts on performance (feed intake, growth and meat yield), mortality, and reproduction [1].

Decades of selection for muscle accretion have resulted in broilers that have excellent performance in economic traits, but do not acclimatize well to stressful environmental conditions such as high temperature and humidity [3–5]. Using traditional breeding methods, it has been difficult to develop broiler genetics for adaptation to climate change while maintaining high performance. High-throughput sequencing technology has accelerated the breeding of livestock through modern breeding methods such as marker-assisted selection. Substantial efforts are underway to identify specific genes associated with tolerance and sensitivity to heat stress [6–9]. High genetic variability between and within breeds suggests that it is feasible to select for tolerance to heat stress [2]. In contrast to broilers, chicken breeds indigenous to hot environments are able to survive in hot conditions, but have poorer production traits. Using chicken lines divergent for heat response will provide contrasting genetic backgrounds to give insights into genes and signaling pathways that are signatures of impacts of heat stress.

The liver plays a variety of roles in energy metabolism, digestion (bile production) and immune capacity. Besides having functions of glycogenolysis and glycogen synthesis, hormone production, and detoxification, the liver is also more susceptible to oxidative stress than other organs under acute heat stress in broilers [10–13]. Heat stress also leads to several changes in physiological responses causing temporary illness to permanent damage that may lead to death [14–16]. The liver is also prone to injury under heat stress and, thus, was an ideal candidate tissue to study the impact of heat stress on organismal energy transformation, hormone metabolism and immune response.

Coble et al. have previously studied the broiler liver transcriptome responses to treatment with cyclic high ambient temperature and observed changes in metabolic, physiologic, and cellular responses [6]. In the present study, we employed high-throughput RNA sequencing (RNA-seq) to conduct liver transcriptome profiling from 3 distinct chicken genetic lines for response to acute and chronic heat stress. RNA-seq provides an unbiased approach that can be used to characterize gene expression changes at the transcriptome level, supporting the maximum reconstruction of an organism’s complex genomic response to heat stress. We also utilized Ingenuity Pathway Analysis (IPA) to help combine the current study’s data with information from other bioinformatics resources to identify genes and pathways associated with a treatment of interest [17]. Our results provide insights into the molecular mechanisms associated with the liver’s responses to 2 different heat stress treatments of 3 distinct chicken lines. The comprehensive examination of the molecular mechanisms underlying heat stress response will assist in the ultimate goal of breeding chickens that are more adapted to high ambient temperatures.

## Methods

### Experimental design

**Fig. 1:**
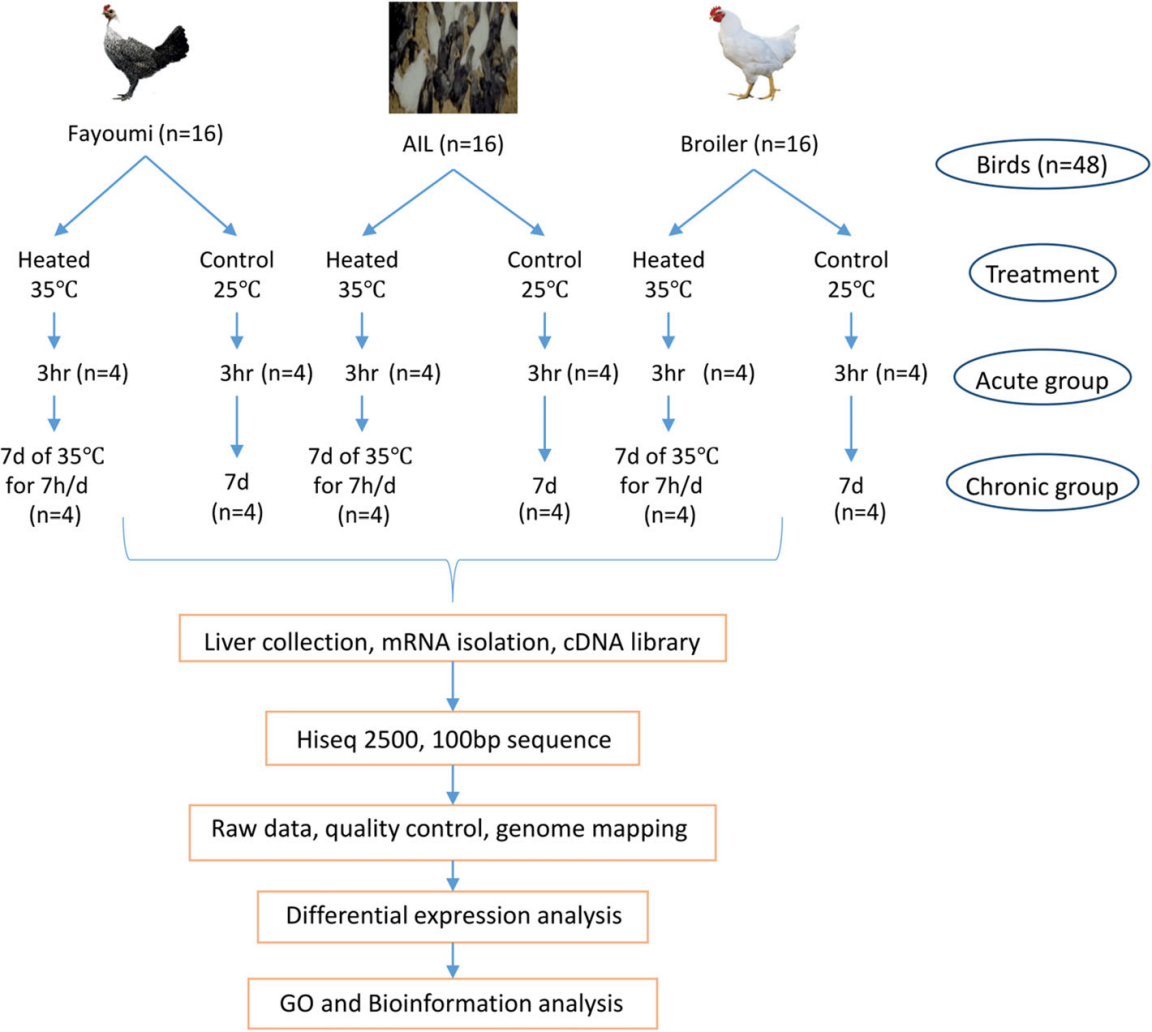
Experiment and analysis design

The 3 distinct genetic lines maintained at Iowa State University that were used in this study were a heat-susceptible broiler line, the heat-resistant Fayoumi, and the F_19_ generation of a highly advanced intercross line (AIL) between the broiler and Fayoumi [8, 18]. The Fayoumi line originated from a hot climate (desert region of Egypt) and has not undergone selection for production traits. This line provides a unique genetic resource for exploring the genes related to heat stress resistance [8]. The AIL of 19 generations of intercrossing between two highly divergent chicken lines (broiler x Fayoumi) were created to help identify and map the genetic loci associated with various traits, including heat tolerance [8]. At 17 days of age, male birds were transferred to temperature-controlled chambers and acclimated to the new environment for five days. Experimental design details were previously published in a report of a study [8] that characterized immune tissue samples collected from this experiment. From 22 to 28 days of age, the heat stress group (*n* = 24) were exposed to high ambient temperature (35 °C) for 7 h per day and remained at 25 °C at all other times. The contemporary control group (*n* = 24) was maintained at 25 °C. Liver samples were collected 3 h after the heat treatment began for the day. The acute heat treatment group (*n* = 12, 4 per line) was euthanized and livers collected on day 1 of heat treatment, and the chronic heat treatment group (*n* = 12; 4 per line) was euthanized and the livers collected on day 7 of cyclic heat (Fig. 1).

### RNA isolation, cDNA library construction and sequencing

Total RNA were isolated from 48 liver samples using RNAqueous® total RNA isolation kit (Ambion, Austin, TX, USA) according to the manufacturer’s protocol. RNA concentration, purity and integrity were first measured with NanoDrop ND-1000 UV-vis Spectrophotometer (NanoDrop Technologies Inc., Wilmington, DE, USA) followed by RNA Nano 6000 Assay kit on Bioanalyzer 2100 system (Agilent Technologies, CA, USA). cDNA libraries were generated from total RNA with Illumina TruSeq® RNA Sample Preparation v2 Kit (Illumina, San Diego, CA, USA) following previously described procedures [19]. Quality of the 48 individuals’ libraries was assessed with the DNA Nano 1000 Assay kit, also on the Bioanalyzer 2100 system. Sequencing was performed at the Iowa State University DNA facility using Illumina® HiSeq 2500 with all 48 samples loaded on a single chip with 6 samples per lane: 4 lanes of 3 h samples and 4 lanes of 7d samples, blocked by treatment and line.

### Sequence reads quality control, mapping, and annotation

Quality of the raw reads was assessed using FastQC (Version 0.10.1) [20]. Adapters and low quality bases were trimmed with fastX clipper (Version 0.0.13) [21]. All 100-bp single-end reads of 48 samples from 12 treatments were separately aligned to the chicken reference genome (*Gallus gallus* 4.0, version 81, Ensembl) using Tophat2. Subsequently, reads were counted by gene using HTseq and *Gallus gallus* genome GTF file (*Gallus gallus* 4.0, version 81, Ensembl). Unless otherwise stated above, all software were run with the default parameters.

### Differential expression gene identification, PCA, gene ontology term

Most of the differentially expressed genes (DEGs) analysis was completed by using a pipeline written in R (Version 3.2.3). DEGs were identified by using the edgeR package (Version 3.12.0) using trimmed mean of M-value (TMM) method for normalization and a generalized linear model (negative binomial) for model fitting [22]. The Benjamini-Hochberg method was applied to control false discovery rate (FDR) at 0.05 [23]. DEGs were considered to be significant for *q*-values <0.05 and log2 fold-changes ≥1. Venn diagrams were created using the limma package (Version 3.26.7). The DESeq2 package (Version 3.12.0) was used to perform principle component analysis (PCA). Gene ontology (GO) analysis was completed with both topGO using adjusted *p-*values and GOseq controlling for gene length. Only the shared GO terms from both GO analyses were considered to be enriched. Additionally, REVIGO was used to summarize and visualize the enriched GO terms [24]. Unless otherwise stated above, all software were run with the default parameters.

### Function annotations, pathway analysis, network analysis

All normalized counts data were uploaded to Ingenuity Pathway Analysis (IPA, Qiagen Redwood City, www.qiagen.com/ingenuity) to identify over-represented canonical pathways based on the Ingenuity® Knowledge Base. Gene names were first matched to the IPA database based on Ensembl ID followed by an attempt to manually match gene names that did not initially match. IPA was used to predict the upstream biological regulators and possible downstream effects on cellular and organismal biology [25]. The threshold of DEGs was set at FDR <0.05 and absolute fold change ≥2. A pathway or function was considered to be active or inhibited when the IPA-predicted absolute z-score was above 2 [25]. Gene networks were also constructed using IPA.

### Fluidigm qPCR validation and statistical analysis

Twenty-four candidate genes related to heat stress response from the 6 heat versus control contrasts were validated with Real-Time qPCR (RT-qPCR). This was performed on a 48x48 dynamic array chip (Fisher Scientific, Pittsburgh, PA) using the Biomark HD system (Fluidigm, San Francisco, CA). A total of 48 RNA samples (representing all 12 treatment groups) in the RNA-seq analysis were simultaneously detected on one array chip. All primer pairs corresponding to the 22 candidate genes and the 2 internal reference genes (GAPDH and RPL4) were designed and synthesized by Fluidigm (see Additional file 1). The efficiency of each primer pair was tested through conventional RT-qPCR on the DNA Engine Opticon® 2 system (BioRad, Hercules, CA) by using the standard curve method [26]. As for accuracy and specificity, only primer pairs with amplification efficiency of more than 0.95 which also gave products with single peaks in melting curve analyses were used for Fluidigm validation. Data produced from Fluidigm RT-qPCR protocol were analyzed based on previously described procedures [27]. Correlation between RNA-seq expression level and Fluidigm RT-qPCR log2 fold change was calculated with Microsoft Excel 2013 (Microsoft, Redmond, WA).

## Results

### Chicken liver transcriptome alignment and mapping

A little more than 1.6 billion, 100-base single-end reads were produced from the single chip run on the Illumina HiSeq 2500 platform. On average, 3.39 gigabases of sequence data were obtained per sample. An average of 83.58% of reads were mapped to the chicken reference genome using Tophat2. Of these reads, samples had an average of 13,915 genes detected, accounting for about 81.33% transcripts of all 17,108 annotated chicken genes. Prior to differential expression analysis, genes were filtered with counts per million (cpm > 1) to eliminate genes with low counts across multiple samples (less than 4 out of the 48 samples) resulting in a final count table with 11,556 genes. A summary of the alignment and mapping results can be found in Additional file 2. Principle component analysis (PCA) results show the 3 genetic lines separated with the AIL located in the middle, between its two parental lines (see Additional file 3). There was also a clear second cluster in the PCA plot, however, it was not the treatment effect. We explored additional factors such as experimental conditions, and technical effects, but no single effect could explain the second cluster. Heat treatment effect was identified in other principle components that explained a smaller percentage of the variation (data not shown). Thus, heat treatment effect is present but less than that of some other factors, such as breed and potentially combinations of other factors.

### Effects of acute heat stress on liver transcriptome of three genetic lines

**Fig. 2:**
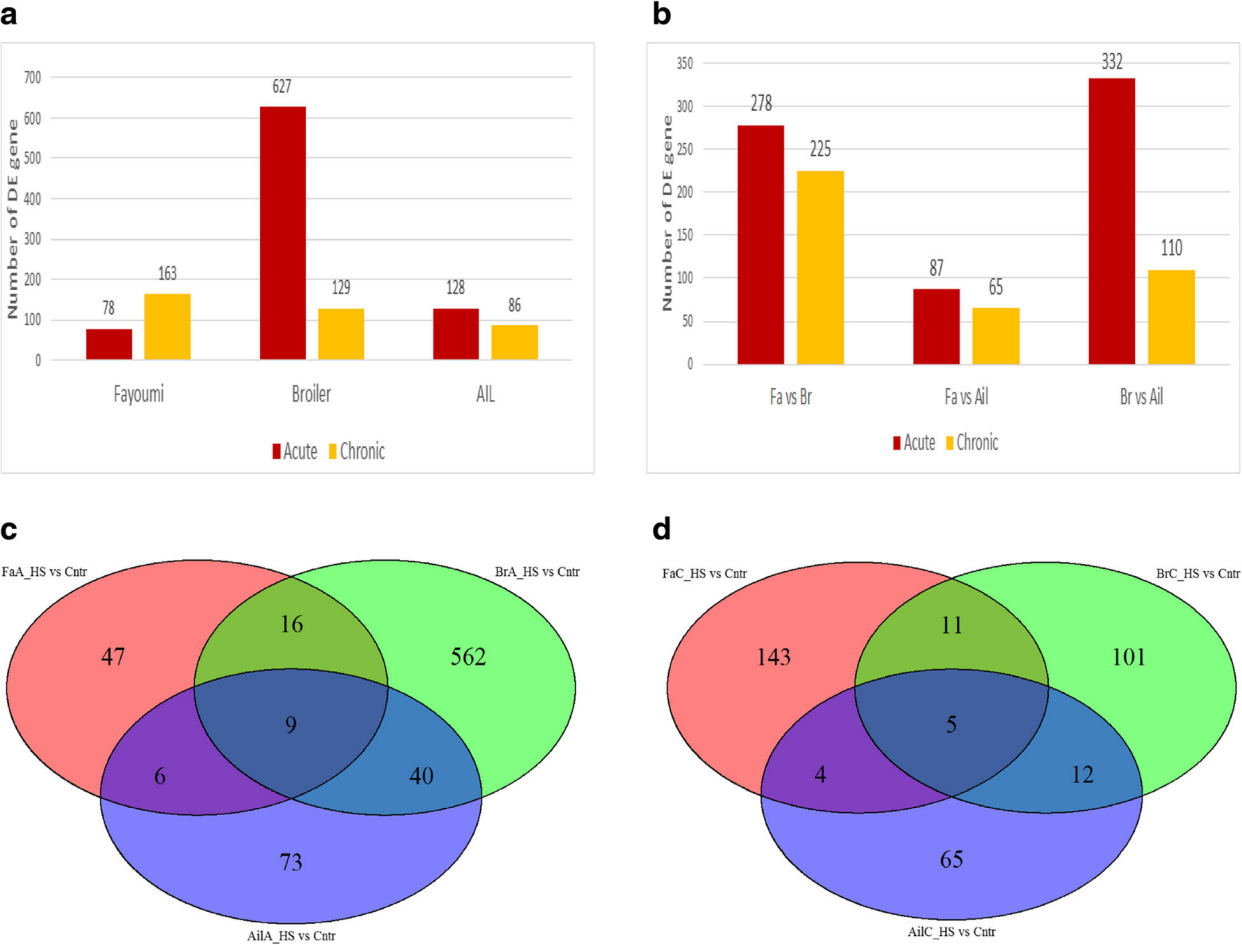
Differentially expressed genes (DEGs) results. **a** Contrasts of heat vs. control samples. There are 8 times more (*n* = 627) differentially expressed genes in broiler acute-heat group compared to control sample than in Fayoumi (*n* = 78). **b** Contrasts between the 3 chicken lines. AIL are more similar to Fayoumi than Broiler in heat stress response. **c** Venn diagram of the acute heat vs. control DEGs for all 3 chicken lines. There are a total of 9 genes found in all the contrasts. **d** Venn diagram of the chronic heat vs. control DEGs for all 3 chicken lines. There are a total of 5 genes found in all the contrasts

**Fig. 3:**
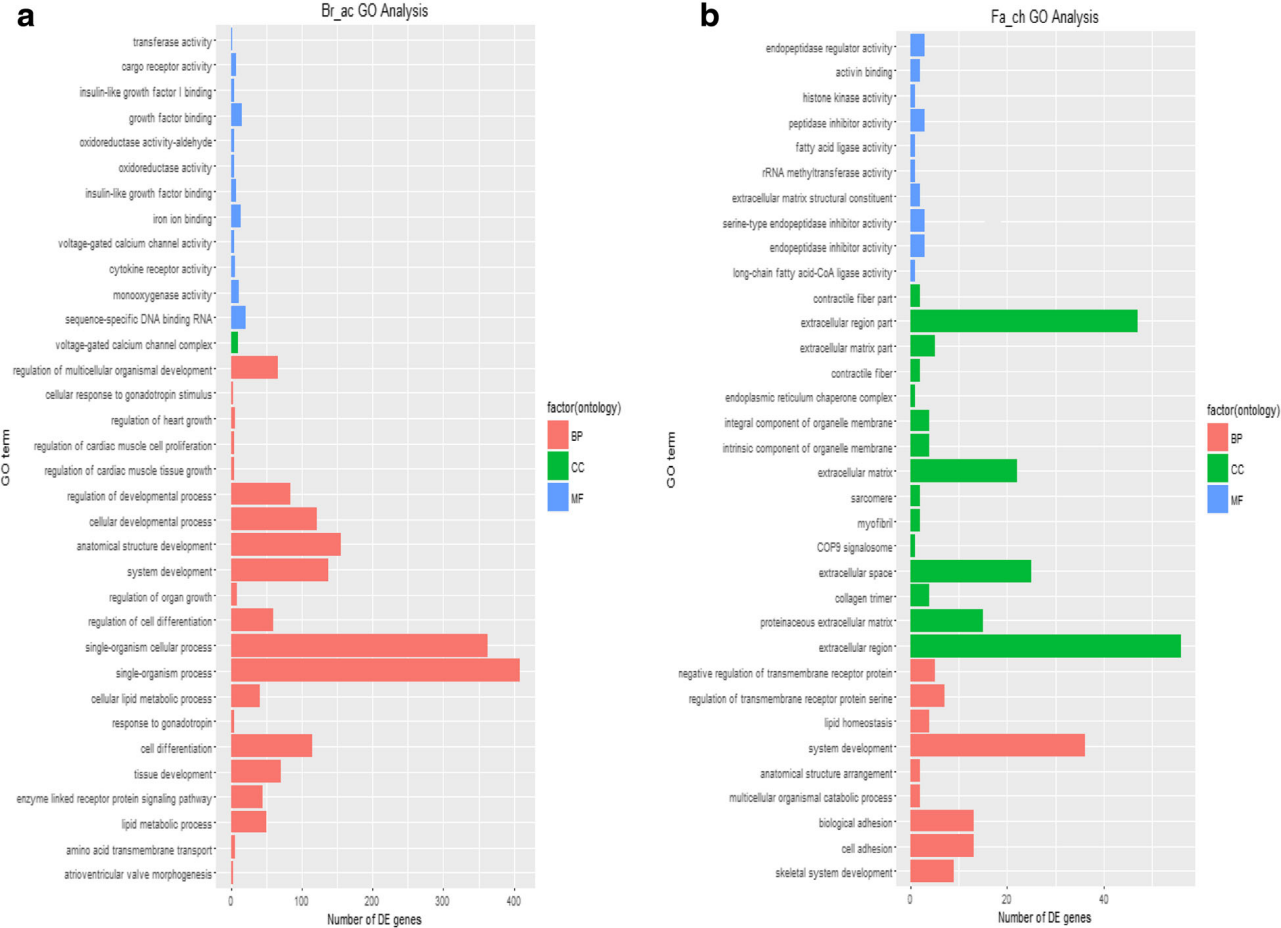
Gene Ontology (GO) analysis output. Biological Process (BP), Cellular Component (CC), and Molecular Function (MF). **a** Broiler acute heat stress GO terms. Most of the terms are in the BP category. **b** Fayoumi chronic heat stress GO terms. Most of the terms are in the CC category

Upon differential expression analysis using normalized read counts, many DEGs between the acute heat stress group and the control group were revealed. Acute heat versus non-heat control contrasts resulted in 8 times more DEGs in broiler (*n* = 627) than in Fayoumi (*n* = 78, Fig. 2a). A small number DEGs (*n* = 25) were shared between the two contrasts (see Additional file 4). From number of DEGs, AIL has a more similar response to heat with Fayoumi than broiler, only having 128 DEGs (Fig. 2a). Of the 128 DEGs found in the AIL contrast, 15 and 49 DEGs were shared with broiler and Fayoumi, respectively, but the expression patterns were not consistent between the lines (see Additional file 4). Across the 3 acute heat versus control contrasts (Fig. 2c), 9 DEGs were observed in all 3 contrasts: angiopoietin-like 4 (ANGPTL4), noggin precursor (NOG), unc-5 homolog A (UNC5A), hexokinase domain containing 1 (HKDC1), transmembrane protein 154 (TMEM154), spindling 1 (SPINW) and 3 uncharacterized genes. GO term analysis of broiler DEGs under acute heat stress revealed 21 of the top 34 significant GO terms were in the biological process (BP) category including: “system/tissue/cellular development”, “response to gonadotropin”, “cell differentiation”, “cellular lipid metabolic process” and “lipid metabolic process” (Fig. 3a). GO terms in the molecular function (MF) category showed genes functioning in “growth factor binding”, “insulin-like growth factor”, “oxidoreductase activity-aldehyde” and “transferase activity”.

### Effects of chronic heat stress on liver transcriptome of three genetic lines

The broiler (*n* = 129) and AIL (*n* = 86) have fewer DEGs between the chronic heat stress group and the control group, than between acute heat stress and control (Fig. 2a). In contrast, the Fayoumi had 163 DEGs under chronic heat stress, more than double the number of DEGs for acute heat stress (78 DEGs, Fig. 2a). The majority of Fayoumi DEGs (63.8%) were up-regulated by chronic heat stress. Five DEGs (FST, SIK1, HKDC1, gadd45, and 1 uncharacterized) were shared among the genetic lines in the 3 chronic heat stress contrasts (Fig. 2d). All shared DEGs between the treatment contrasts can be found in Additional file 4. Fifteen of the top 34 significant GO terms from Fayoumi chronic heat stress contrast were in the cellular component (CC) category enriched in “contractile fiber part”, “extracellular region/matrix”, “integral/intrinsic component of organelle membrane” and “myofibril” (Fig. 3b). Compared to the acute group, GO terms in the BP category showed genes enriched in “negative regulation of transmembrane receptor protein”, “lipid homeostasis”, “skeletal system”, “biological adhesion” and “cell adhesion” (Fig. 3b).

### Pairwise comparison between three genetic lines’ response to heat stress in liver

When contrasting Fayoumi and broiler under acute heat stress, 278 DEGs were identified (Fig. 2b). As shown in Additional file 5, DEGs with the largest FCs included growth arrest and DNA damage-inducible protein (GADD), hexokinase domain containing (HKDC), angiopoietin-like 4 (ANGPTL4), NADPH oxidase 1 (NOX1) and lysyl oxidase-like 4 (LOXL4). During acute heat stress, there were more DEGs between AIL and broiler (*n* = 332) than AIL and Fayoumi (*n* = 87, Fig. 2b). Similarly, for chronic heat stress, a larger number of DEGs were observed between AIL and broilers (*n* = 110) compared to AIL and Fayoumi (*n* = 65, Fig. 2b).

Using REVIGO on GO terms generated from contrasting broiler and Fayoumi response to acute heat stress, 19 GO terms categories were revealed. These categories included “positive regulation of response to stimulus”, “organic acid metabolism” and “immune system process” (see Additional file 6A). Only 8 enrichment GO terms categories were identified by REVIGO between broiler and Fayoumi lines under chronic heat stress. These categories included “response to external stimulus”, “defense response”, and “single-organism cellular process” (see Additional file 6B).

### Pathway analysis of 3 genetic lines under heat stress in liver

**Fig. 4:**
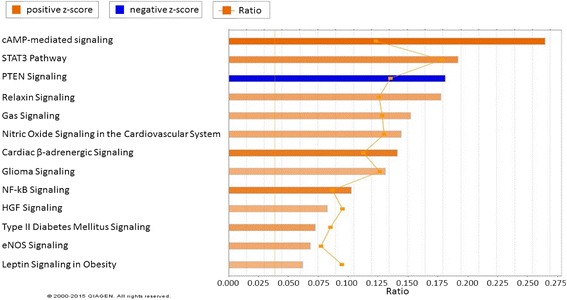
Functional analysis of signaling pathway expression in broiler acute vs. control contrast from IPA. The pathways with the most significant change in acute heat stress are shown. The more orange the bar in the chart, the greater the activity. In contrast, the more blue the bar in the chart, the greater the inhibition. Heat stress caused changes in the cardiovascular system (Pathways: Nitric Oxide Signaling in the Cardiovascular System and Cardiac B-adrenergic Signaling), nervous system (Pathway: Glioma Signaling), immune system (Pathways: PTEN Signaling and NF-kB Signaling), hepatic system (Pathways: HGF Signaling and Leptin Signaling in Obesity), and circulatory system (Pathways: eNOS Signaling)

**Fig. 5:**
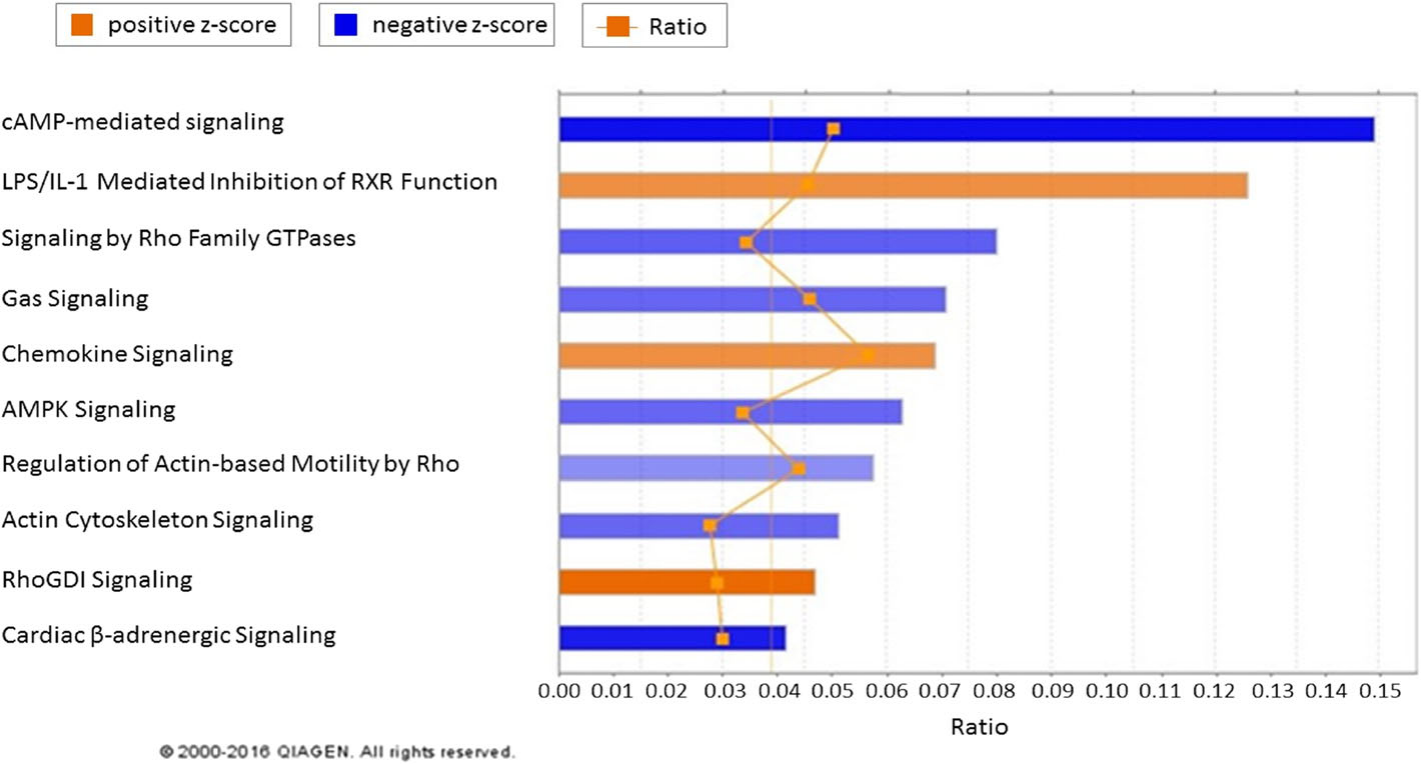
Functional analysis of signaling pathway expression in Fayoumi vs. Broiler under acute heat contrast from IPA. The pathways with the most significant change in acute heat stress are shown. The more orange the bar in the chart, the greater the activity. In contrast, the more blue the bar in the chart, the greater the inhibition. Heat stress caused more inhibition of pathways in Fayoumi when compared to Broiler

Canonical pathway analysis in IPA for broiler acute heat treatment showed that all 13 enriched pathways were activated, with the exception of PTEN signaling (Fig. 4). All the activated pathways were involved in essential biological systems: cardiovascular system (Nitric Oxide Signaling in the Cardiovascular System and Cardiac B- adrenergic Signaling), nervous system (Glioma Signaling), immune system (NF-kB Signaling), hepatic system (HGF Signaling and Leptin Signaling in Obesity), and circulatory system (eNOS Signaling). Canonical pathway analysis was also performed between Fayoumi and broiler under acute heat stress, and 7 of 10 enriched pathway were inhibited (Fig. 5).

Downstream analysis of biological functions based on DEGs from Fayoumi and broiler contrasts under acute and chronic heat stress identified top functional terms of “quantity of cells”, “metabolism of terpenoid and steroid”, “organismal death” and “angiogenesis” (see Additional file 7).

### Network analysis of 3 genetic lines under heat stress

**Fig. 6:**
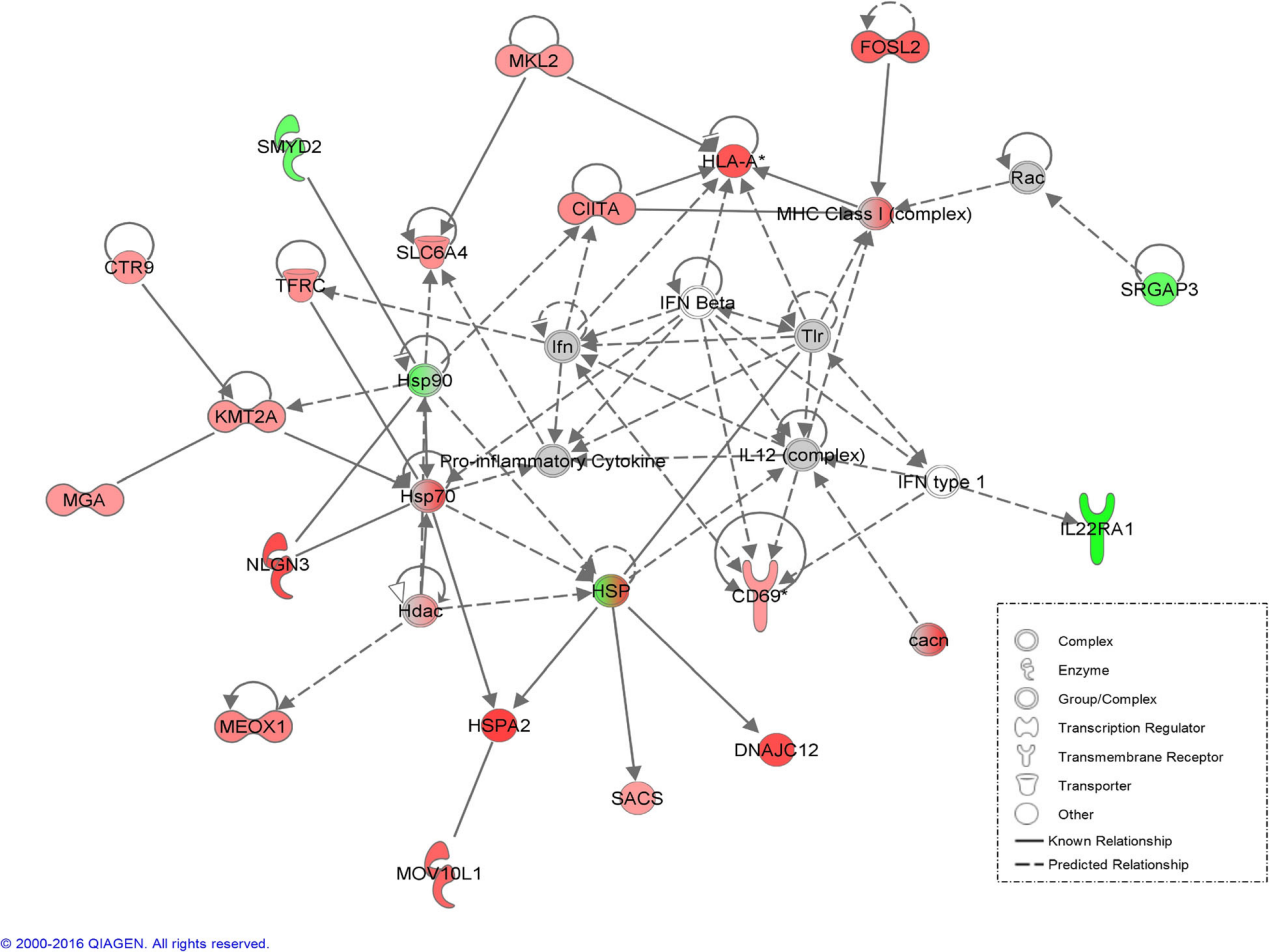
Network analysis for the differentially expressed genes (DEGs) of acute heat stress response in Broiler. Network shows numerous connections between heat shock proteins (HSP, Hdac, Hsp70, Hsp90, HSPA2, DNAJC12) and immune related genes (MHC Class I, HLA-A, IFN, Proinflammatory Cytokine, IFN Beta, TLR, IL12, IFN type 1, and IL22RA1). Dotted lines are indirect interactions and solid lines are direct interactions. Genes in red were up-regulated and in green were down-regulated. The grey genes were not DEGs and white genes were not expressed but predicted by IPA to be involved in the network

A novel interaction network involved in broiler acute heat stress response was generated with IPA (Fig. 6). The network predicted interactions between many genes from the heat shock protein gene family (HSP, Hdac, Hsp70, Hsp90, HSPA2, DNAJC12) and immune related genes (MHC Class I, HLA-A, IFN, IFN Beta, TLR, IL12, IFN type 1, and IL22RA1). These included many of the DEGs. Most of the heat shock protein genes (except HSP 90) and immune related genes (except IL22RA1) were up-regulated. Also from IPA, “Lipid Metabolism” and “Organismal Injury, Abnormalities and Inflammatory Response” were the two highest ranking networks in all 3 genetic lines (data not shown).

### Validation of RNA-seq data by Fluidigm

Fluidigm qPCR system was used to validate the expression level for 24 genes of interest. These genes were selected based on average expression level in RNA-seq data (FPKM > 1), heat stress and immune response function, and primer performance (see Additional file 1). Correlation of 0.883 between the RNA-seq and Fluidigm qPCR log2 fold change was observed for the acute broiler vs. control broiler contrast (see Additional file 8).

## Discussion

Numerous studies have reported the detrimental effects of high ambient temperature on physiological, biochemical, and immune capacity of chickens [5, 28–32]. It has been proposed that heat stress impairs mitochondrial functions and induces oxidative damage in plasma, liver, heart and skeletal muscle of broilers [5]. Varasteh et al. found differential gene expression of broiler intestine to heat stress [9], and Zuo et al. identified a reduction in skeletal muscle protein deposition in response to high ambient temperature [33]. In addition to metabolic effects, altered immune response of broilers under heat stress has also been widely reported [9, 14, 34–39]. However, little is known about the transcriptome regulation of chickens from distinct genetic lines to both acute and chronic heat stress. In a previous study of the broiler liver response to chronic high ambient temperature stress, metabolic and physiologic changes were partly characterized using an RNA-seq approach [6]. In the current study, we further explored the mechanism of heat stress in three genetic lines: heat-susceptible (broiler), heat-resistant (Fayoumi) and the F_19_ AIL of these two parental lines. Moreover, we analyzed the response from 2 heat treatments: acute heat stress for 3 h and chronic heat stress for 7d with cyclic heat. This created a complex experimental design that produced many interesting comparisons and contrasts between the 12 treatment groups. These comparisons contributed to the identification of candidate genes and affected pathways related to heat stress response.

### Broiler, Fayoumi and AIL heat stress response at the gene level

PCA results showed that the 3 genetic lines had distinct responses under heat stress. Broilers showed a considerably stronger response to heat stress at the gene regulatory level, with a much higher number of DEGS than the other two lines, suggesting a line difference in heat sensitivity and the mechanisms to alleviate acute heat stress. There were very few overlapping DEGs between the AIL and either parental line, and the number of DEGs between lines suggest the AIL responds to heat in a manner more similar to the Fayoumi and the broiler. The shared DEGs for both acute and chronic heat stress identified across all 3 genetic lines may potentially representing important candidate genes that are common to all chickens in the molecular response of chicken to heat stimulation. Specifically, the ANGPTL4 is an angiogenic factor that has been recognized as a central player in various aspects of energy homoeostasis and regulation of reactive oxygen species (ROS) [40]. Consistent with a prior study, ANGPTL4 was also found to be down-regulated in the heat versus control contrast of the more heat-susceptible broiler breed [6]. However, opposite result (up-regulated) was observed for the more heat-resistant Fayoumi breed in both acute and chronic heat versus control contrasts. This helps to emphasize the key role that ANGPLT4 plays in the function of liver metabolic changes in response to heat condition. With further confirmation, ANGPLT4 can potentially serve as a selection target to improve heat resistance for the broiler breed. GO analysis in broiler showed DEGs enrichment in the organism and cell development, energy metabolic and hormone regulation, suggesting heat stress is impairing growth and development, while Fayoumi and AIL had not observed this phenomenon. Taken together, the results demonstrated that there are clearly differences in responses for the 3 genetic lines under heat stress, but common mechanisms for heat response also exist.

### Comparing the Fayoumi and broiler lines’ response to heat stress

Heat stress has different effects on the body temperature, heterophil:lymphocyte ratio, and plasma corticosterone concentration in distinct breeds [41]. Van Goor et al. used an AIL developed from broiler and Fayoumi to identify quantitative trait loci for body temperature, production traits and blood chemistry under heat stress [8, 18]. We compared Fayoumi in heat stress to broiler under heat stress after contrasting each line’s heat treatment group against the control group. The top significant up-regulated genes involved angiopoietin (ANGPTL4), DNA damage (*gadd*), and oxidases (NADPH oxidase and LOXL4). The expression of ANGPTL4 was higher in Fayoumi than Broiler, which further supported its key role in heat response for chickens. Additionally, Ropp et al. reported that *gadd* was expressed as a DNA damaging reagent under heat and oxidative stress [42]. Ito et al. demonstrated that the *gadd* promoter was activated by the expression of the TNF-α gene [43]. The *gadd-*TNF-α system also causes a cytotoxic response that is effective in killing tumor cells [43]. During pathway analysis, we found Chemokine Signaling and RhoGDI signaling pathways were active in Fayoumi heat stress response when compared to broiler heat stress response. The RhoGDI family of proteins plays a negative role in Rho-family GTP-dissociation, which has functions in cytokinesis, phagocytosis and cell motility, and control Rho protein homeostasis. RhoGDI also reduced epithelial cell integrity and increased permeability for humans with chronic inflammation [44]. Similarly, the GO results showed Fayoumi have different responses than broilers in heat stress. The responses involved sensitivity to stimulus, metabolism changes, and immune capacity. The comparison of heat stress responses for different genetic lines helped explore the effects of the genome on regulation mechanisms.

### Comparing the acute vs. chronic heat stress response

Acute heat stress caused a stronger response than chronic heat stress in heat-susceptible broilers. The acute contrast (acute heat stress versus control) compared to the chronic contrast (chronic heat stress versus control) resulted in 4.8 times more DEGs in the broiler. Comparing chronic with acute heat treatment, there are many DEGs in each of the 3 genetic lines: Fayoumis (*n* = 339), Broiler (*n* = 419) and AIL (*n* = 241). This implies that all lines utilize different response mechanisms to acute versus chronic heat exposure. A previous study reported that acute heat stress primarily caused a disturbance of plasma metabolites, whereas chronic heat stress resulted in tissue damage [30]. Compared to acute heat stress, chronic heat stress did not induce oxidative damage due to a probable self-propagating scavenging system [31]. Mashaly et al. found that production and reproduction performance of laying hens were significantly reduced, and immune functions were inhibited in the acute heat stress group compared to the chronic heat stress group [45]. We found similar results in the Fayoumi, which inhibited immune response in acute compared to chronic heat stress and an opposite expression pattern in the broiler. Almost all the physiologic, metabolic, and immune functions were significantly active under acute heat stress, but these same functions were inhibited when the heat stress lasted for several days. Exploration of common biological processes is needed to adapt chickens to both acute and chronic heat stress through genetic selection.

### Functional analysis of signaling pathway expression

Numerous researchers have proposed that apoptosis is promoted under heat stress [35, 46–51]. Apoptosis may play a vital role in the physiology or pathophysiology of heat-related illness attributed to heat stress [36]. Moulin et al. reported that mild heat shock stimulated TNF-related apoptosis, inducing ligand-mediated apoptosis of leukemic T lymphocytes and promyelocytic cells [46]. In our results, TNF was the top activated regulator in both broiler and Fayoumi acute heat stress broiler contrast. This consistency between the 2 distinct genetic lines lends support to the hypothesis that heat stress induces an inflammatory response in order to reduce mortality. TNF can potentially serve as a heat stress response marker.

Heat shock proteins (HSPs) were reported to function in the suppression of proapoptotic signaling. This suppression may be directly or by stabilizing elements of the NF-kB pathway to promote cellular survival [40]. Many studies have established a role of heat shock proteins on immune response [48, 52–58]. The IPA network generation tool produced an interesting gene network that combined heat shock proteins and immune-related genes. Heat shock response is ubiquitous as an essential defense mechanism for protection of cells from a wide range of harmful conditions, including heat shock, alcohols, heavy metals, oxidative stress, fever, infection and/or inflammation [59]. A regulatory function of heat shock proteins is to activate the immune system by providing danger signals in order to down-regulate immune and inflammatory response by acting as signaling receptors for stress cytokines [51, 60, 61]. The gene expression results were consistent with the IPA network analysis. HSP70, HSPA2, DNAJC12, HDAC were highly expressed in the broiler acute heat stress contrast, whereas genes critical to immune function, IFN, IFN α/β, IL12, TIR, were lowly expressed and had no significant difference. Only HSP90 was down-regulated; it is not a stress response protein, but rather a constitutive molecular chaperone involved in surface expression of the Toll-like receptors [60]. The information from this network analysis elucidated genes and/or potential pathways that utilize heat shock protein activities to modify protein expression. The purpose of this response may be to alleviate the effects of heat stress.

## Conclusions

This study, which examined heat stress responses of 3 distinct chicken genetic lines, has provided numerous insights into the effects of an environmental stressor on organismal metabolism and the immune signaling pathways that initiate repair, allow adaptation, and ensure survival. Based on transcriptome expression levels, RNA-seq analysis identified a stronger response from acute heat stress compared to chronic heat stress. The analysis also showed that broilers have the strongest transcriptome response to high ambient temperature among the 3 lines. The newly characterized candidate genes and networks involved in the response to heat might serve as a foundation for future contemporary selection breeding for the purpose of improving the chicken’s ability to better handle heat stress.

## Additional files

Additional file 1: Primer information for the 24 genes used in Fluidigm qPCR validation of RNA-seq results. (DOCX 16 kb)
Additional file 2: Basic information on sequencing output and processing. (DOCX 17 kb)
Additional file 3: PCA plot of mRNA expression as predicted from RNA-seq data. (TIF 32 kb)
Additional file 4: List of shared differentially expresses genes among 3 genetic lines (XLSX 16 kb)
Additional file 5: List of differentially expressed genes with the largest fold changes from the Fayoumi chronic heat vs. Broiler chronic heat contrast (DOCX 15 kb)
Additional file 6: REVIGO analysis of GO terms enrichment. (A) Fayoumi vs. Broiler contrast under acute heat and (B) Fayoumi vs. Broiler contrast under chronic heat. (PNG 797 kb)
Additional file 7: Heat-map of downstream regulators. (PNG 504 kb)
Additional file 8: Correlation between RNA-seq and Fluidigm qPCR. (PNG 297 kb)
