# Supplementary material for: Liver transcriptome response to hyperthermic stress in three distinct chicken lines
Source: BMC Genomics. 2016 Nov 22;17:955. doi: 10.1186/s12864-016-3291-0 (PMC5118885; doi:10.1186/s12864-016-3291-0)
Supplement: Additional file 1: — Primer information for the 24 genes used in Fluidigm qPCR validation of RNA-seq results. (DOCX 16 kb) [file 12864_2016_3291_MOESM1_ESM.docx]

**Additional file 1.** Primer information for the 24 genes used in Fluidigm qPCR validation of RNASeq results.

| Ensembl ID | Gene | Design RefSeq | FPKM | Forward Primer | Reverse Primer |
| --- | --- | --- | --- | --- | --- |
| ENSGALG00000016200 | SIK1 | NM_204682.1 | 8.879545462 | GAGCAGCAGAGAGGGAAGAAA | GGAGTAGCTGGTGATGCTGAA |
| ENSGALG00000012726 | HSP90B1 | NM_204289.1 | 531.7480561 | AGTAGAGAAGGGTCCCGAAC | GGATGCATTTAGGCCATCCA |
| ENSGALG00000002634 | HDAC3 | NM_204747.1 | 13.12771122 | AACAACATGCAGGGCTTCAC | ACTCAAAGAGCCCTGGAAACA |
| ENSGALG00000003297 | HDAC1 | NM_204156.1 | 11.75577566 | GAGTACAGCAAGCAGATGCA | ACTCAAACAGCCCATCGAAC |
| ENSGALG00000004288 | HDAC4 | NM_204313.1 | 3.520048356 | CTGGTTTTGACGCAGTGGAA | TGCTTCGTCAGGTACCCAAA |
| ENSGALG00000014991 | HDAC2 | NM_204831.2 | 23.37088609 | CGGCAAGAAGAAAGTCTGCTAC | TCATTGGATGCCCTTGTCCATA |
| ENSGALG00000001320 | CYP1A1 | NM_205146.2 | 126.0030533 | TTGTTGCAGACAGCTGTGGAA | TCGATGAGGGAGTCGGTGAC |
| ENSGALG00000014501 | FGFBP2 | NM_204447.1 | 2.770994517 | TCATGCCTGCGAATCTACACA | TTGCTTCATGAGGGCATCCA |
| ENSGALG00000027901 | THRSP | NM_213577.2 | 14.48569893 | AGGAGATCAACCTCGGTGAA | TGGGACTTGGCACAGGAATA |
| ENSGALG00000010866 | AREG | NM_001031537.1 | 1.650600242 | GCATCCATGGTGAATGTGTGTAC | ACTGTTCACCACAGCGTTCA |
| ENSGALG00000014908 | FST | NM_205200.1 | 7.411775229 | AGCCCGAACTTGAAGTCCAATA | GAGCTGCCTGGGCATAAAAC |
| ENSGALG00000017077 | HSPH1 | NM_001159698.1 | 8.531239945 | GTAGTTTCGTTCGGCTCCAA | CTGTGTTGTGGGCATGAGTAA |
| ENSGALG00000019201 | HSPA13 | NM_001030793.2 | 6.567560165 | GCAACGGAATGCCACTGTTA | GCAGCTGTGGGTTCATTGATTA |
| ENSGALG00000001000 | HSPA5 | NM_205491.1 | 473.1819932 | TTTCTGCCATGGTCCTGACAA | AGGCTGGCACAGTAACAACA |
| ENSGALG00000001926 | HSPB1 | NM_205290.1 | 5.566309027 | ATCCCCGAGGATTGGTACAA | GCAGCAGACGGAAGTATCC |
| ENSGALG00000002363 | HSPA9 | NM_001006147.1 | 98.24496004 | GTTCATGTGTCAGCCAAGGAC | TCTTTGCTAAGGCCACCAGAA |
| ENSGALG00000006512 | HSPA8 | NM_205003.2 | 333.15451 | CTGGCAAGGAGAACAAGATCAC | TTCCTGAACCATCCGCTCAA |
| ENSGALG00000008094 | HSPD1 | NM_001012916.1 | 218.2212006 | TGAAGTTTGACCGAGGCTACA | GACGTAAGCATCCTGGAATTCAC |
| ENSGALG00000026137 | CDKN2B | NM_204433.1 | 12.5299992 | ACGGCTGCGGATGAACTA | CCGTCCAGTAGCTCCTTCA |
| ENSGALG00000010185 | HSPA4L | NM_001012576.1 | 20.76949398 | CCTCTAAGAGCAGCCATGGAA | TTCTAGTAGCCCCACCGACTA |
| ENSGALG00000011351 | HSP90AA1 | NM_001109785.1 | 408.5010035 | ACACATGCCAACCGCATTTA | CCTCCTCAGCAGCAGTATCA |
| ENSGALG00000007861 | MAGT1 | NM_001006435.1 | 33.91508502 | GAAGGCTCGGATGTGTTTCA | AGGCTTCCCTTTAGCAGGAA |
| ENSGALG00000014442 | GAPDH | NM_204305.1 | 4.787384146 | GTGCTGGCATTGCACTGAA | CACAACACGGTTGCTGTATCC |
| ENSGALG00000007711 | RPL4 | NM_001007479.1 | 28.41462891 | TTCTGCCTTGGCAGCATCA | AGGAAGTTCTGGGATCTCCTCA |
