# Supplementary material for: Liver transcriptome response to hyperthermic stress in three distinct chicken lines
Source: BMC Genomics. 2016 Nov 22;17:955. doi: 10.1186/s12864-016-3291-0 (PMC5118885; doi:10.1186/s12864-016-3291-0)
Supplement: Additional file 2: — Basic information on sequencing output and processing. (DOCX 17 kb) [file 12864_2016_3291_MOESM2_ESM.docx]

**Additional file 2.** Basic information on sequencing output and processing

| **Sample** | Total reads | Tophat2 mapped | Mapped percentage | Genes with counts | Transcriptome % | Population | Lane | RIN |
| --- | --- | --- | --- | --- | --- | --- | --- | --- |
| **1** | 33091050 | 27638836 | 83.50% | 13694 | 80.04% | Broiler | 8 | 8.6 |
| **3** | 30870036 | 26109376 | 84.60% | 14131 | 82.60% | Fayoumi | 5 | 9.2 |
| **32** | 30683070 | 24698590 | 80.50% | 13749 | 80.37% | Fayoumi | 6 | 8.6 |
| **54** | 36159636 | 30083591 | 83.20% | 13910 | 81.31% | Broiler | 5 | 8.7 |
| **110** | 30256003 | 26071194 | 86.20% | 14118 | 82.52% | Broiler | 6 | 8.5 |
| **197** | 37170495 | 31075527 | 83.60% | 14108 | 82.46% | AIL | 5 | 8.8 |
| **292** | 38821859 | 31236228 | 80.50% | 13868 | 81.06% | AIL | 6 | 8.9 |
| **2102** | 31133488 | 25009734 | 80.30% | 14390 | 84.11% | Fayoumi | 1 | 8.2 |
| **2103** | 34061053 | 27687085 | 81.30% | 13887 | 81.17% | Fayoumi | 6 | 8.8 |
| **2105** | 36285429 | 28342938 | 78.10% | 13937 | 81.46% | Fayoumi | 7 | 9.1 |
| **2112** | 37147556 | 31251691 | 84.10% | 13959 | 81.59% | Fayoumi | 7 | 8.6 |
| **2113** | 41711363 | 36213532 | 86.80% | 14202 | 83.01% | Fayoumi | 2 | 9.6 |
| **2115** | 29569363 | 25203090 | 85.20% | 13595 | 79.47% | Fayoumi | 2 | 8.8 |
| **2117** | 36606856 | 30495721 | 83.30% | 13954 | 81.56% | Fayoumi | 3 | 8.9 |
| **2120** | 40832636 | 34431654 | 84.30% | 13965 | 81.63% | Fayoumi | 5 | 8.9 |
| **2131** | 32764897 | 27554698 | 84.10% | 13753 | 80.39% | Fayoumi | 8 | 8.7 |
| **2134** | 30652722 | 25952640 | 84.70% | 13892 | 81.20% | Fayoumi | 8 | 8.5 |
| **2138** | 36726454 | 30348204 | 82.60% | 13937 | 81.46% | Fayoumi | 4 | 9.2 |
| **2141** | 33287738 | 26871812 | 80.70% | 13801 | 80.67% | Broiler | 5 | 8.5 |
| **2145** | 35011452 | 27168190 | 77.60% | 13645 | 79.76% | Broiler | 1 | 8.6 |
| **2162** | 40521361 | 35240492 | 87.00% | 13985 | 81.75% | Broiler | 1 | 8.5 |
| **2163** | 26336573 | 22335931 | 84.80% | 13953 | 81.56% | Broiler | 2 | 8.0 |
| **2169** | 31506201 | 27118199 | 86.10% | 14074 | 82.27% | Broiler | 3 | 8.9 |
| **2170** | 35135285 | 29499080 | 84.00% | 13935 | 81.45% | Broiler | 3 | 7.9 |
| **2174** | 32237147 | 27639110 | 85.70% | 13947 | 81.52% | Broiler | 6 | 8.3 |
| **2200** | 31743244 | 27112763 | 85.40% | 13573 | 79.34% | AIL | 1 | 9.5 |

**Additional file 2.** Basic information on sequencing output and processing (cont.)

| **Sample** | Total reads | Tophat2 mapped | Mapped percentage | Genes with counts | Transcriptome % | Population | Lane | RIN |
| --- | --- | --- | --- | --- | --- | --- | --- | --- |
| **2203** | 36016913 | 30079512 | 83.50% | 13963 | 81.62% | AIL | 7 | 8.9 |
| **2206** | 32927086 | 28221002 | 85.70% | 14031 | 82.01% | AIL | 1 | 7.6 |
| **2228** | 35335889 | 30598576 | 86.60% | 14188 | 82.93% | AIL | 2 | 8.4 |
| **2237** | 28481069 | 24092779 | 84.60% | 13973 | 81.68% | AIL | 5 | 8.3 |
| **2242** | 39536593 | 29412794 | 74.40% | 13973 | 81.68% | AIL | 6 | 9.3 |
| **2315** | 31365029 | 26659489 | 85.00% | 14103 | 82.44% | AIL | 3 | 8.0 |
| **2339** | 32948034 | 28253539 | 85.80% | 13828 | 80.83% | AIL | 4 | 8.5 |
| **2379** | 35451681 | 30151659 | 85.10% | 13732 | 80.27% | Fayoumi | 1 | 8.0 |
| **2399** | 33499414 | 28621078 | 85.40% | 13875 | 81.10% | Fayoumi | 4 | 9.1 |
| **2404** | 33222743 | 27815314 | 83.70% | 13771 | 80.49% | Fayoumi | 3 | 8.4 |
| **2438** | 36725976 | 30184883 | 82.20% | 13895 | 81.22% | Broiler | 7 | 8.1 |
| **2443** | 35023166 | 29876368 | 85.30% | 13897 | 81.23% | Broiler | 2 | 7.4 |
| **2455** | 33218326 | 28454291 | 85.70% | 13800 | 80.66% | Broiler | 4 | 8.0 |
| **2465** | 22069446 | 17470545 | 79.20% | 13425 | 78.47% | Broiler | 4 | 8.6 |
| **2467** | 34613130 | 28246409 | 81.60% | 13914 | 81.33% | Broiler | 4 | 8.3 |
| **2482** | 36764782 | 29559721 | 80.40% | 14237 | 83.22% | Broiler | 8 | 8.4 |
| **2517** | 30157626 | 26155056 | 86.70% | 13776 | 80.52% | AIL | 4 | 8.2 |
| **2528** | 30567716 | 24760259 | 81.00% | 13905 | 81.28% | AIL | 8 | 8.5 |
| **2543** | 37608626 | 31787680 | 84.50% | 14037 | 82.05% | AIL | 8 | 9.3 |
| **2559** | 36393195 | 31925642 | 87.70% | 14057 | 82.17% | AIL | 2 | 7.9 |
| **2561** | 33147477 | 27880528 | 84.10% | 13791 | 80.61% | AIL | 7 | 8.4 |
| **2567** | 34453816 | 29341580 | 85.20% | 13818 | 80.77% | AIL | 3 | 8.3 |
