# Supplementary figures and images for: Liver transcriptome response to hyperthermic stress in three distinct chicken lines
Source: BMC Genomics. 2016 Nov 22;17:955. doi: 10.1186/s12864-016-3291-0 (PMC5118885; doi:10.1186/s12864-016-3291-0)

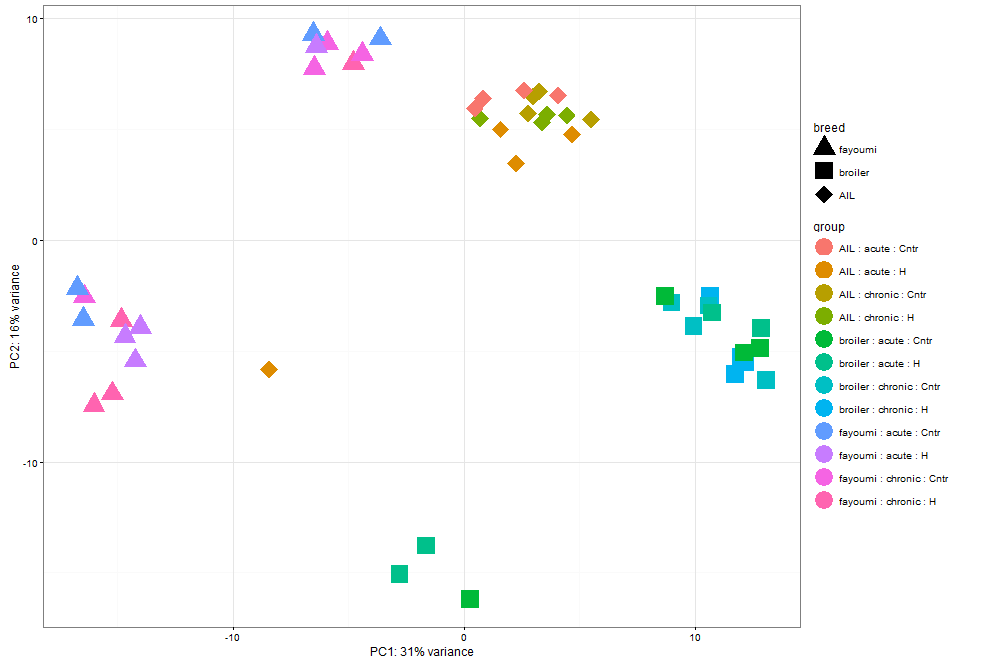

Supplement: Additional file 3: — PCA plot of mRNA expression as predicted from RNA-seq data. (TIF 32 kb) [file 12864_2016_3291_MOESM3_ESM.tif]

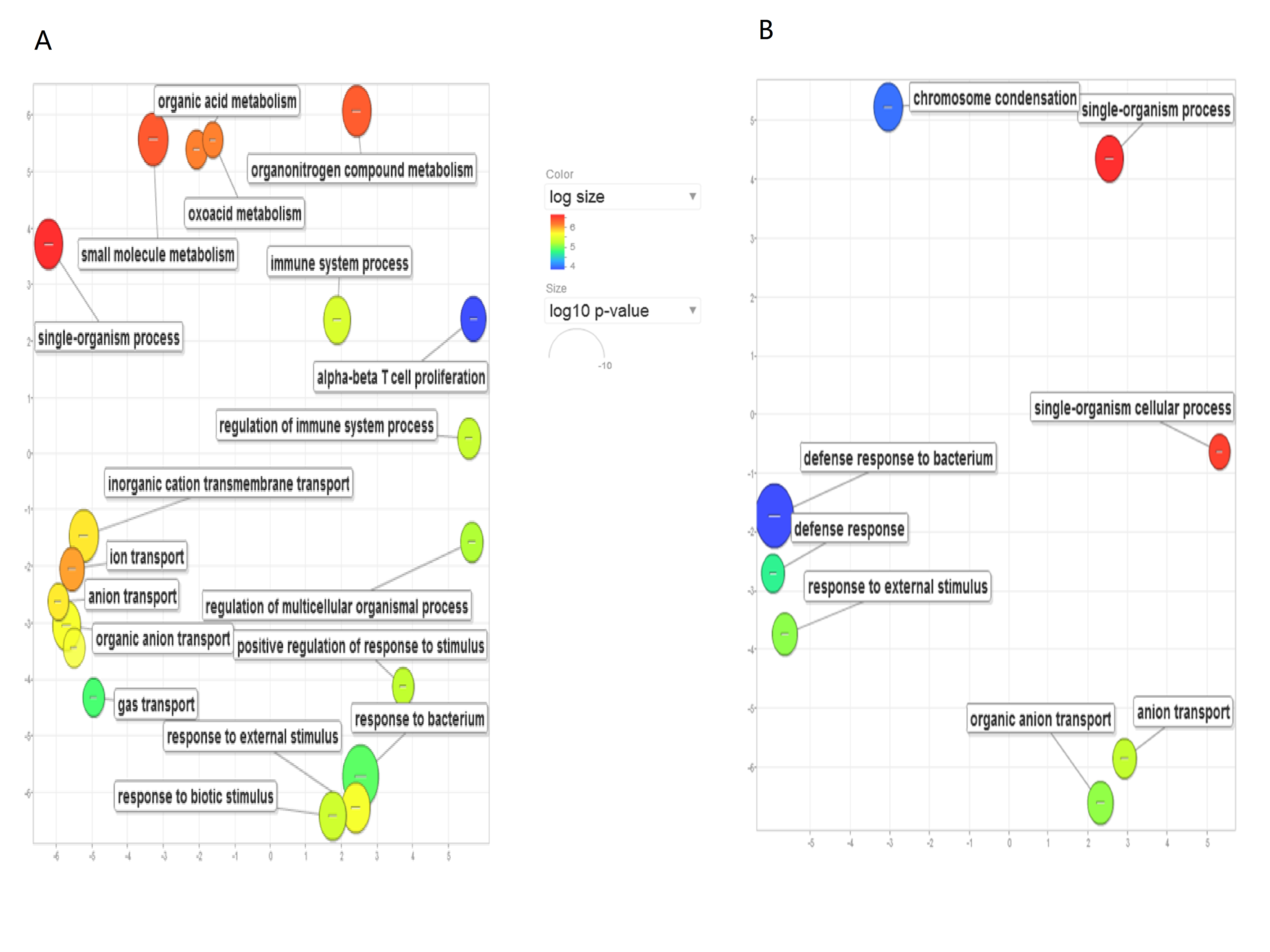

Supplement: Additional file 6: — REVIGO analysis of GO terms enrichment. (A) Fayoumi vs. Broiler contrast under acute heat and (B) Fayoumi vs. Broiler contrast under chronic heat. (PNG 797 kb) [file 12864_2016_3291_MOESM6_ESM.png]

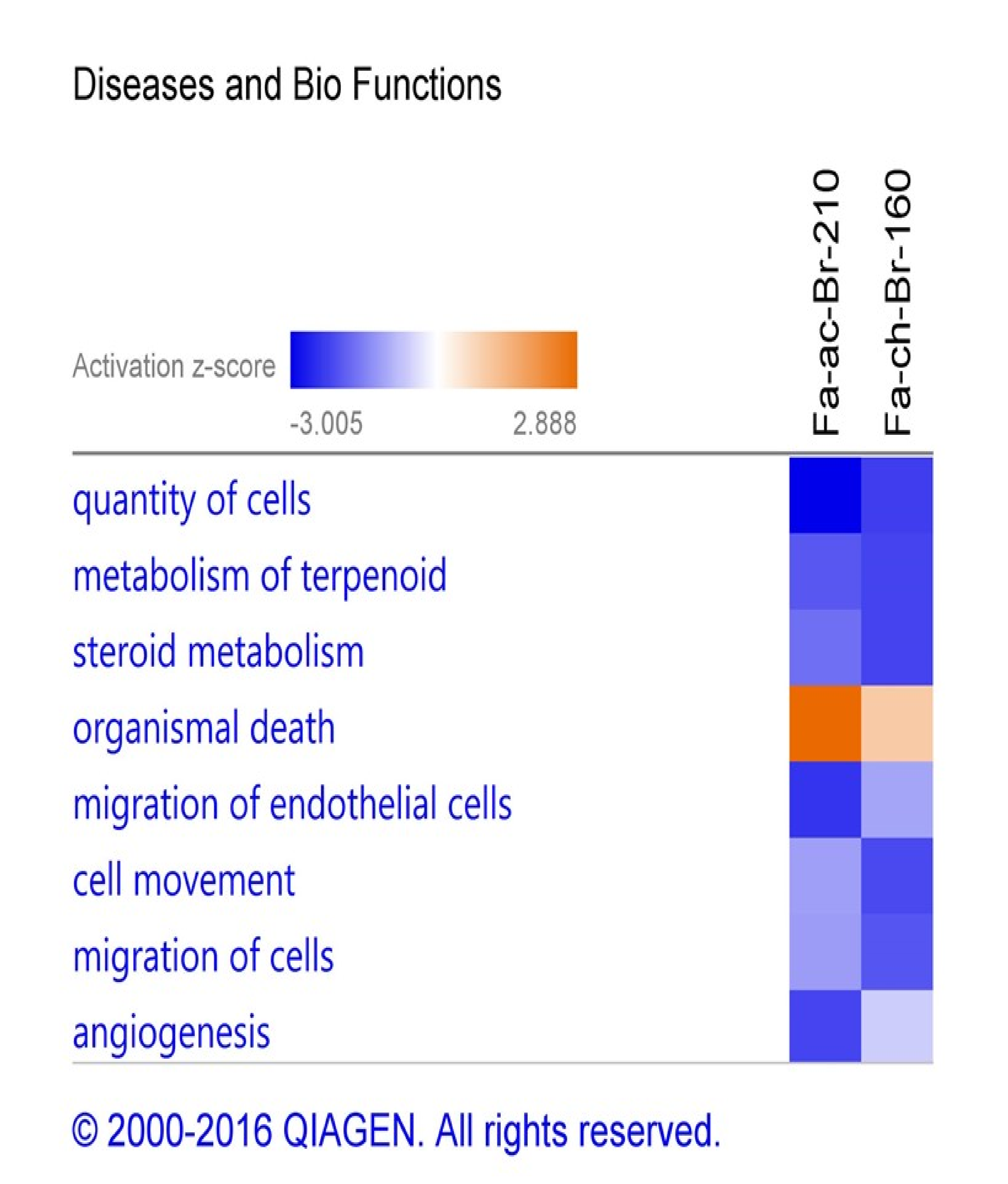

Supplement: Additional file 7: — Heat-map of downstream regulators. (PNG 504 kb) [file 12864_2016_3291_MOESM7_ESM.png]

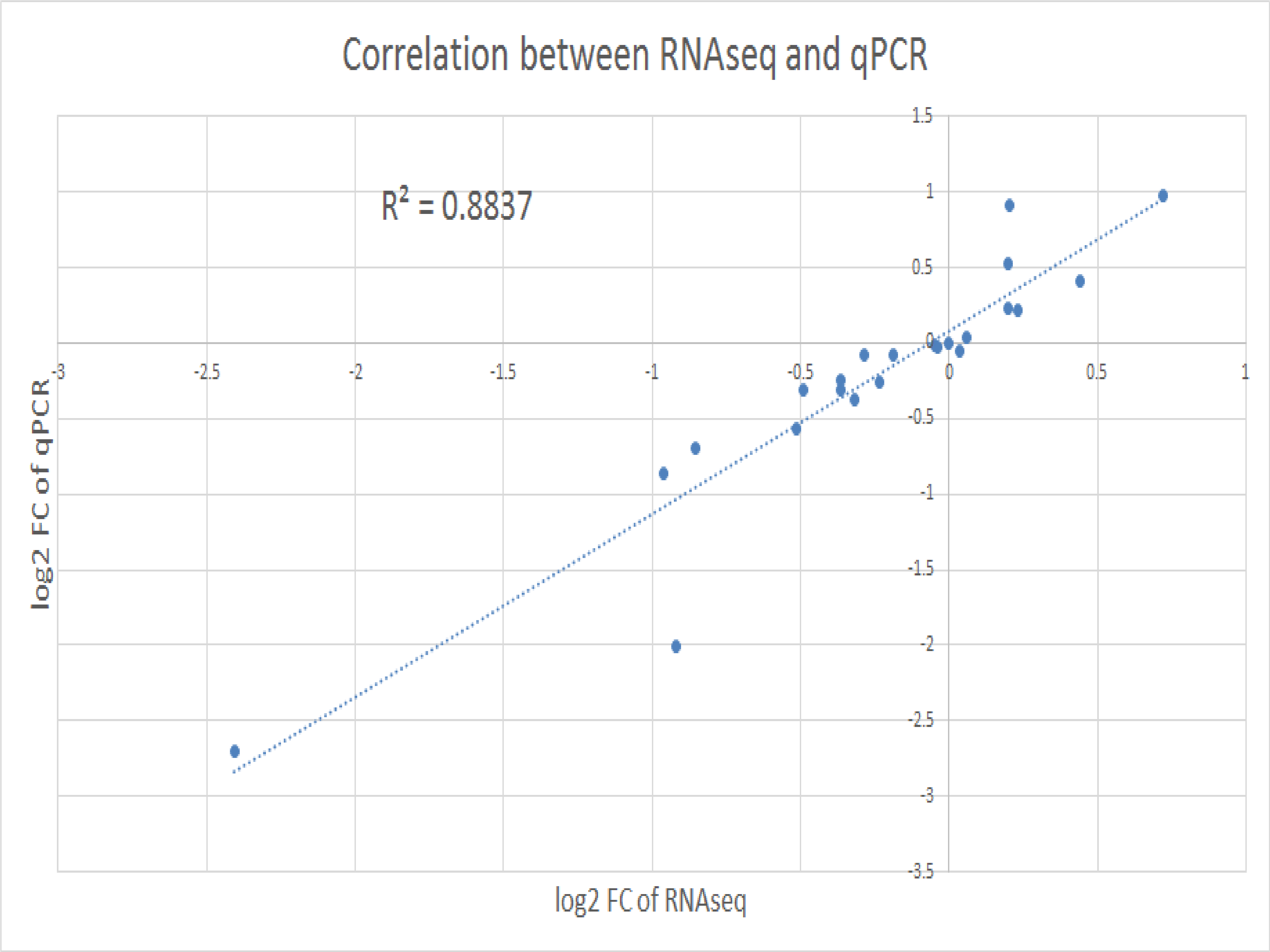

Supplement: Additional file 8: — Correlation between RNA-seq and Fluidigm qPCR. (PNG 297 kb) [file 12864_2016_3291_MOESM8_ESM.png]
