# Supplementary material for: Liver transcriptome response to hyperthermic stress in three distinct chicken lines
Source: BMC Genomics. 2016 Nov 22;17:955. doi: 10.1186/s12864-016-3291-0 (PMC5118885; doi:10.1186/s12864-016-3291-0)
Supplement: Additional file 5: — List of differentially expressed genes with the largest fold changes from the Fayoumi chronic heat vs. Broiler chronic heat contrast (DOCX 15 kb) [file 12864_2016_3291_MOESM5_ESM.docx]

| Gene ID | logFC | FDR | Gene Name | Description |
| --- | --- | --- | --- | --- |
| ENSGALG00000004703 | 5.326505 | 8.84E-07 |  | Uncharacterized protein |
| ENSGALG00000028005 | 5.320142 | 3.88E-36 | gadd45 | growth arrest and DNA damage-inducible protein GADD45 gamma |
| ENSGALG00000021039 | 4.665636 | 6.11E-29 | HKDC1 | hexokinase domain containing 1 |
| ENSGALG00000006644 | 4.481885 | 6.70E-20 | ACSL6 | acyl-CoA synthetase long-chain family member 6 |
| ENSGALG00000000619 | 4.453515 | 1.25E-26 | ANGPTL4 | angiopoietin-like 4 |
| ENSGALG00000011354 | 4.160301 | 0.006976 | CRYBA2 | beta-crystallin A2 |
| ENSGALG00000006922 | 4.078502 | 2.87E-09 |  | Uncharacterized |
| ENSGALG00000010439 | 3.903264 | 7.02E-14 |  | Uncharacterized |
| ENSGALG00000023355 | 3.87405 | 3.47E-18 | TMEM154 | transmembrane protein 154 |
| ENSGALG00000003114 | 3.784767 | 3.53E-11 | NOG | noggin precursor |
| ENSGALG00000012254 | 3.529444 | 3.12E-07 | cKir2.3 | Uncharacterized |
| ENSGALG00000001749 | 3.437321 | 4.56E-16 | ACSBG2 | long-chain-fatty-acid--CoA ligase ACSBG2 |
| ENSGALG00000023933 | 3.370094 | 4.47E-15 | G0S2 | G0/G1switch 2 |
| ENSGALG00000006707 | 3.072409 | 2.54E-10 | NOX1 | NADPH oxidase 1 |
| ENSGALG00000013090 | 2.881848 | 0.021273 | LOXL4 | lysyl oxidase-like 4 |
| ENSGALG00000017184 | 2.76837 | 0.00011 | MMP7 | matrilysin precursor |
| ENSGALG00000003134 | 2.76221 | 0.00603 |  | Uncharacterized |
| ENSGALG00000006637 | 2.757566 | 6.65E-10 |  | XK-related |
| ENSGALG00000018998 | 2.712082 | 8.46E-07 | UNC5A | unc-5 homolog A |
| ENSGALG00000026597 | 2.571323 | 0.010543 |  | Uncharacterized |
| ENSGALG00000015822 | 2.524681 | 1.18E-07 | MAP3K7CL | MAP3K7 C-terminal like |
| ENSGALG00000010825 | 2.518616 | 0.057204 | AGR2 | anterior gradient 2 |
| ENSGALG00000015944 | 2.442945 | 7.35E-07 | RIMS1 | regulating synaptic membrane exocytosis 1 |
| ENSGALG00000008233 | 2.335282 | 2.78E-07 | BUB1 | mitotic checkpoint serine kinase BUB1 |
| ENSGALG00000000769 | 2.318538 | 0.001739 | RAB7B | RAB7B |
| ENSGALG00000029160 | 2.283225 | 0.005831 | LRRC4 | leucine rich repeat containing 4 |
| ENSGALG00000012396 | 2.271936 | 0.000276 | PTGER2 | prostaglandin E2 receptor EP2 subtype |
| ENSGALG00000012367 | 2.265198 | 1.71E-06 | TRIM9 | tripartite motif containing 9 |
| ENSGALG00000023172 | 2.256272 | 0.009443 |  | Uncharacterized |

**Additional file 5.** List of differentially expressed genes with the largest fold changes from the Fayoumi chronic heat vs. Broiler chronic heat contrast
